# Association Between Serum HBV DNA Levels and CCL-20, CD8a, CXCL-16, and GDF-15 in Patients with Chronic Hepatitis B

**DOI:** 10.3390/v17101352

**Published:** 2025-10-08

**Authors:** Burak Ezer, Hilal Sena Esen, Selin Ugrakli, Mehmet Sinan Iyisoy, Mehmet Ozdemir

**Affiliations:** 1 Medical Microbiology Laboratory, Beyhekim Training and Research Hospital, University of Health Sciences, 42090 Konya, Turkey; 2 Department of Medical Microbiology, Faculty of Medicine, Necmettin Erbakan University, 42090 Konya, Turkey; hciftci1996@gmail.com (H.S.E.); dr.selinyumakci@gmail.com (S.U.); 3 Department of Medical Education and Informatics, Faculty of Medicine, Necmettin Erbakan University, 42090 Konya, Turkey; siyisoy@gmail.com; 4 Division of Medical Virology, Department of Medical Microbiology, Faculty of Medicine, Necmettin Erbakan University, 42090 Konya, Turkey; mehmetozdem@yahoo.com

**Keywords:** chronic hepatitis B, HBV DNA, CCL-20, CD8a, CXCL-16, GDF-15

## Abstract

The aim of our study is to determine the changes in the biomarkers CXCL-16, CCL-20, GDF-15, and CD8a, which play an immunological role in CHB patients according to viral load to determine their diagnostic potential and to investigate their relationships with hematological parameters and non-invasive fibrosis indices. Our study included 96 chronic hepatitis B patients and 30 healthy individuals as a control group. The patients were divided into three groups based on their serum HBV DNA levels: mild (0–10^2^ IU/mL), moderate (10^3^–10^5^ IU/mL), and severe viral load (10^6^–10^8^ IU/mL). HBV DNA levels were determined by the real-time PCR (Anatolia, Istanbul, Turkey) method. CXCL-16, GDF-15, and CD8a levels in patient serum were quantitatively determined by the ELISA method (Elabscience, Wuhan, China), and CCL-20 levels were determined by the ELISA method BT LAB, Shanghai, China). ROC (Receiver Operating Characteristics) and HUM (Hypervolume Under Manifold) analyses were used to determine the diagnostic efficacy of the biomarkers. ROC analyses showed that GDF-15 (AUC = 0.920) and CCL-20 (AUC = 0.751) had “very good” and “good” diagnostic values, respectively, in predicting hepatitis B disease. HUM analyses revealed that all biomarkers have good potential when it comes to distinguishing the severity of the disease. This study has shown that the biomarkers GDF-15 and CCL-20 may be potential diagnostic biomarkers in detecting the presence of chronic hepatitis B, and the biomarkers CXCL-16, CCL-20, GDF-15, and CD8a may be potential diagnostic biomarkers in determining the severity of the disease. These findings suggest that these biomarkers, which can be measured by the simpler and more economical ELISA method, could be a supportive tool for the HBV DNA test. The clinical use of these biomarkers can be expanded with future prospective studies.

## 1. Introduction

The hepatitis B virus (HBV) causes chronic infection in approximately 250 million people worldwide. This infection is a significant global public health problem that can lead to serious complications such as cirrhosis and hepatocellular carcinoma, resulting in significant morbidity and mortality. HBV itself does not have a direct cytopathic effect. The primary mechanism causing liver damage is the host immune system’s response to the virus, which is insufficient to eliminate it [1]. Chronic HBV infections occur as a result of the host immune response’s inability to clear the virus following acute HBV infection. They are generally observed at higher rates in the perinatal period [2]. Chronic HBV infection is based on covalently closed circular DNA (ccDNA), which forms a stable, small chromosomal structure in infected hepatocytes and serves as a permanent template for viral replication [3]. The interplay between the innate and adaptive immune systems is important in the immunopathogenesis of chronic HBV infection. During the acute phase of infection, the innate immune response is typically suppressed or bypassed by the virus [1]. Consequently, the adaptive immune response becomes the primary factor in determining the course of the infection. CD8+ cytotoxic T lymphocytes play a particularly crucial role in viral clearance by eliminating virus-infected hepatocytes [3]. In acute HBV infections, the virus-specific T cell response is strong, polyclonal, and directed against multiple epitopes. Conversely, in chronic HBV infections, the T cell response is weak and narrow-spectrum [3]. This T cell response dysfunction is characterized by loss of T cell proliferation and cytokine production capacity following chronic antigen stimulation. This is one of the main mechanisms of viral persistence [4]. Given these circumstances, chronic HBV infection can be considered an immunodeficiency disease. This emphasizes the importance of investigating biomarkers that reflect both the viral load and the host’s immunological status.

International guidelines recommend measuring HBV DNA quantitatively as the primary parameter in the clinical management of chronic HBV infection in order to determine the disease stage, guide decisions on antiviral therapy, and monitor the patient’s response to treatment [5]. Although HBV DNA testing provides crucial information as a key indicator of viral replication, it has significant limitations. As it is a molecular test, the high cost and need for advanced laboratory infrastructure limit its use in low- and middle-income countries, where the seroprevalence of the disease is highest [6]. Furthermore, serum HBV DNA levels may not always accurately reflect pathological stages such as necroinflammation and fibrosis occurring within the liver. Even when HBV DNA levels are low or undetectable, particularly in HBeAg-negative patients or “inactive carriers”, serious complications such as liver fibrosis and cirrhosis can still develop [7]. Similarly, in occult HBV infection, low-level viral replication may persist in the liver despite HBsAg negativity, leading to serious complications such as fibrosis and hepatocellular carcinoma [8]. Furthermore, quantitatively determined serum HBV levels may not always correlate perfectly with intrahepatic cccDNA, which is the true center of viral replication [9]. Discrepancies between viral replication and the host’s pathological response can complicate the clinical management of the disease. Therefore, there is a need for non-invasive immunological biomarkers that can reflect the immunopathological mechanisms underlying hepatic inflammation and fibrosis and that can be a supportive tool for molecular tests (PCR). Chemokines are molecules that regulate the migration and settlement of immune cells at sites of infection and inflammation. They play a central role in the pathogenesis of chronic liver diseases [10]. CXCL-16 is a chemokine found in transmembrane and soluble forms. It promotes the migration and adhesion of lymphocytes, particularly natural killer T (NKT) lymphocytes that express the CXCR6 receptor, to the liver. In inflamed liver cells, the expression of CXCL-16 increases in sinusoidal endothelial cells and cholangiocytes. Studies have shown that CXCL-16 plays a direct role in hepatic inflammation and fibrosis [11]. CCL-20 is the sole ligand for the CCR6 receptor. It is responsible for the chemotaxis of many cells involved in the immune system, such as Th17, regulatory T cells (Treg), and dendritic cells [12]. CCL-20 levels increase significantly in response to inflammatory stimuli in the liver. It has been reported that CCL-20 levels are significantly increased in various chronic liver diseases, such as alcoholic hepatitis and non-alcoholic fatty liver disease (NAFLD), and that CCL-20 triggers both inflammation and fibrosis in the liver [13]. GDF-15 is a stress cytokine belonging to the TGF-β superfamily. GDF-15 expression increases in various cellular stress conditions, such as inflammation, oxidative stress, hypoxia, and tissue damage. It has been reported in the literature that GDF-15 levels correlate with the severity of liver fibrosis and are significantly elevated in the serum of patients with complications such as cirrhosis and hepatocellular carcinoma arising from chronic HBV infections [14]. CD8+ T cells play a paradoxical role in the immunopathogenesis of chronic HBV infection. While they are critical for viral clearance in acute infection, they become functionally dysfunctional in chronic infection [3]. This dysfunction is the most important mechanism leading to viral persistence [15]. Soluble CD8a (sCD8a) is released into the serum as a result of CD8+ T cell activation and cycling. It may indicate an ongoing but ineffective T cell response in the liver [16]. Therefore, sCD8a levels could reflect the dynamics of the host’s immune response to the virus. In light of the limitations of HBV DNA testing and the pivotal role played by immune-mediated inflammation and fibrosis in chronic HBV infection progression, there is a need for more accessible, less costly, non-invasive biomarkers that reflect the underlying pathological processes. Our study aimed to quantify serum CCL-20, CD8a, CXCL-16, and GDF-15 levels in patients with chronic HBV infection using the enzyme-linked immunosorbent assay (ELISA), which is cheaper, more accessible, and easier to use than molecular methods. Additionally, we will examine the relationship between the quantitative levels of CCL-20, CD8a, CXCL-16, and GDF-15 determined in our study and HBV DNA, investigating their diagnostic accuracy in determining the presence and severity of chronic HBV infection according to viral load, as well as examining their relationships with hemolytic parameters, indices, and non-invasive fibrosis indices.

## 2. Materials and Methods

### 2.1. Ethics Committee and Project Support Information

This scientific research project was evaluated by the Non-Drug and Non-Medical Device Research Ethics Committee of the Faculty of Medicine Hospital at the University of Necmettin Erbakan on 7 June 2024. It was approved for ethical consideration under decision number 2024/5019. The study (project number 24GAP18013) was supported by the Scientific Research Project Coordinatorship of the University of Necmettin Erbakan.

### 2.2. Study Population

The study included 96 patients diagnosed with chronic hepatitis B, with HBsAg detected in their serum for more than six months. The patients were collected from various departments at the Medical Microbiology Laboratory of the University of Necmettin Erbakan University Faculty of Medicine Hospital. The patients’ HBV DNA levels were determined using real-time PCR (Anatolia, Istanbul, Turkey). While grouping the patients, studies in the literature were reviewed and in addition to viral load and HBV DNA values, which are biopsy indications and show a significant increase in mortality, ALT, AST values, ultrasonography findings, and non-invasive fibrosis indices were also taken into account [17]. Patients were divided into three groups according to their HBV DNA levels: 31 patients with levels between 10^0^ and 10^2^; 31 patients with levels between 10^3^ and 10^5^; and 34 patients with levels between 10^6^ and 10^8^. These groups were classified as having mild, moderate, and heavy viral loads. In total, 30 individuals without hepatitis markers (HBsAg and anti-HCV), liver disease (e.g., Wilson’s disease, haemochromatosis, alcoholic/toxic hepatitis, or autoimmune hepatitis), elevated ALT/AST enzymes, or chronic/infectious diseases were selected as the control group. Serum samples were stored at −80 °C until the study was conducted, which used ELISA to quantitatively determine the levels of CD8a, CXCL-16, GDF15 (Elabscience, Wuhan, China) and CCL-20 (BT LAB, Shanghai, China). The inclusion and exclusion criteria are listed in Table 1.

### 2.3. Statistical Analysis

For numerical variables, mean and standard deviation or median (Q1–Q3) values are given, and for categorical variables, frequency and percentage values are given. Chi-squared or Fisher’s exact tests were used to analyze categorical variables, while ANOVA was used to analyze the numerical variables. Tukey’s CLD notation was used for pairwise comparisons. Spearman’s correlation test was used to investigate relationships between numerical variables. A two-category ROC (Receiver Operating Characteristics) analysis was performed for disease diagnosis, and a three-category ROC analysis was performed for degree of disease diagnosis. AUC (Area Under the Curve) and HUM (Hypervolume Under Manifold) values were used for this purpose. All analyses were performed using R 4.4.2 (R Core Team, 2024), with *p* < 0.05 considered significant. HUM analysis is used to evaluate the ability of a single biomarker or a combination of biomarkers to correctly classify disease stages (e.g., mild, moderate, severe). HUM is a generalization of the traditional AUC, which is typically applied to binary situations (e.g., patient vs. control). When the number of classes increases, HUM provides a single measure that reflects the overall discriminatory power across all categories. Instead of conventional ROC curves, HUM relies on a higher-dimensional surface, called the ROC manifold, and calculates the hypervolume under this manifold. This hypervolume serves as an extension of the AUC, allowing for the assessment of diagnostic performance in multi-class settings [18].

## 3. Results

The age and gender distribution of the patients, the descriptive characteristics of hematological parameters and indices and non-invasive fibrosis indices, as well as the relationship between the relevant parameters and indices and viral load, CXCL-16, CCL-20, GDF-15, and CD8a are given in Table 2**.**

The CD8a levels in the light viral load group were found to be statistically significantly higher than in the control group (*p* = 0.004). The GDF-15 levels in the severe viral load group were found to be significantly higher than those in both the control group and the mild viral load group (*p* < 0.001, *p* = 0.011). Similarly, the CXCL-16 levels in the mild viral load group were found to be significantly higher than those in the control group (*p* = 0.009). The CCL-20 levels were statistically significantly higher in the severe and mild viral load groups than those in the control group (*p* < 0.001). The CCL-20 values in the severe viral load group were also significantly higher than those in the moderate and mild viral load groups (*p* < 0.001). Furthermore, the CCL-20 levels were found to be higher in the severe viral load group than in the moderate group (*p* = 0.001).

Spearman’s correlation test was used to examine the correlations of hematological parameters and indices with non-invasive fibrosis indices and between biomarkers. Statistically significant positive correlations were detected between CD8a and CXCL-16 (r = 0.25), between CXCL-16 and GDF-15 (r = 0.25), between CCL-20 and CD8a (r = 0.18), and between CCL-20 and GDF-15 (r = 0.32).

**Figure 1:**
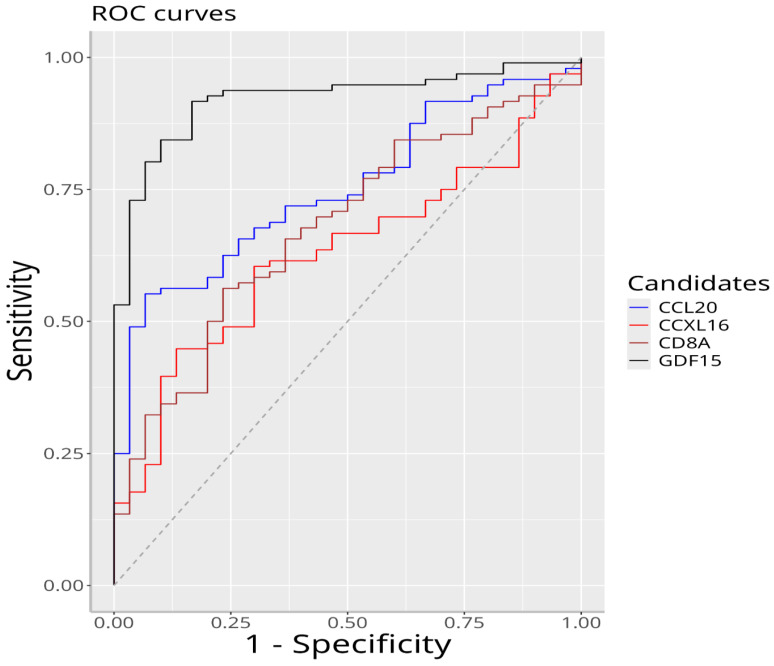
ROC curves of CCL-20, CXCL-16, CD8a, and GDF-15 biomarkers.

We investigated the diagnostic properties of quantitative values of the biomarkers CCL-20, CXCL-16, CD8a, and GDF-15 in predicting hepatitis B using ROC curve analysis (Figure 1). Where significant breakpoints were present, sensitivity and specificity values were calculated. When evaluating the area under the curve, values between 1.00 and 0.90 were considered excellent, those between 0.90 and 0.80 were considered good, those between 0.80 and 0.70 were considered moderate, those between 0.70 and 0.60 were considered poor, and those between 0.60 and 0.50 were considered unsuccessful.

ROC analysis revealed that the CCL-20 values had good statistically significant diagnostic value for predicting hepatitis B in patients (*p* < 0.001, AUC = 0.751). The optimal cutoff value was 183.946, with a sensitivity of 0.552 and a specificity of 0.933.

Similarly, ROC analysis showed that GDF-15 values had excellent statistically significant diagnostic value in predicting hepatitis B (*p* < 0.001, AUC = 0.920). The optimal cutoff value was 391.908, with a sensitivity of 0.917 and a specificity of 0.833.

ROC analysis showed that CXCL-16 values had poor but statistically significant diagnostic value for predicting hepatitis B in patients (*p* < 0.05, AUC = 0.629). The optimal cutoff value was 11.250, with a sensitivity of 0.448 and a specificity of 0.867.

The results of the evaluation using ROC analysis revealed that CD8a values had weak but statistically significant diagnostic value in predicting hepatitis B disease in patients (*p* < 0.05, AUC = 0.678). The optimal cutoff value was 21.593, with a sensitivity of 0.562 and a specificity of 0.767 (Table 3). 

The sensitivity, specificity, and AUC values of CCL-20, GDF-15, CXCL-16, and CD8a are presented in Table 4.

HUM values were used to assess the ability of biomarkers to detect the severity of chronic hepatitis B. Biomarkers with HUM values above 0.16 are considered capable of distinguishing the severity of hepatitis B disease effectively.

**Figure 2:**
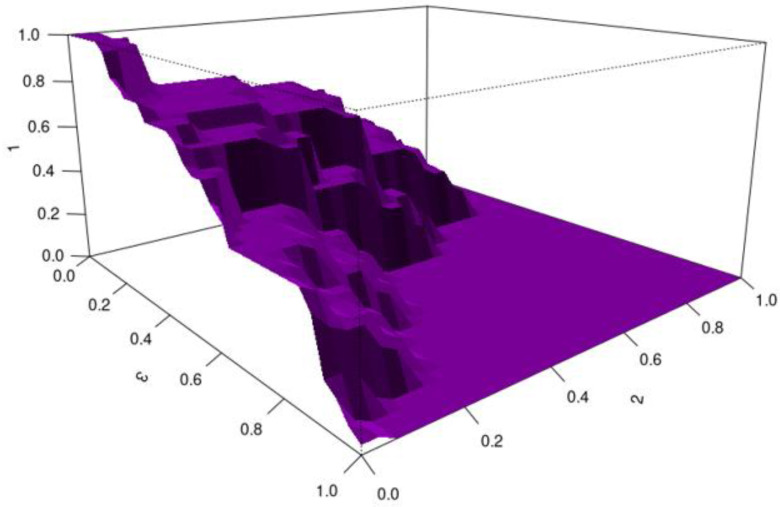
HUM image for CD8a (X-axis: CD8a quantitative values; Y-axis: HUM value).

For CD8a, the HUM value is 0.245 and the cutoff value is 11.33–24.391. CD8a was found to be an effective biomarker for distinguishing disease severity in patients with chronic hepatitis B, with quantitative values up to 11.33 indicating mild disease, values between 11.33 and 24.391 indicating moderate disease, and values of 24.391 and above indicating severe disease (Figure 2).

**Figure 3:**
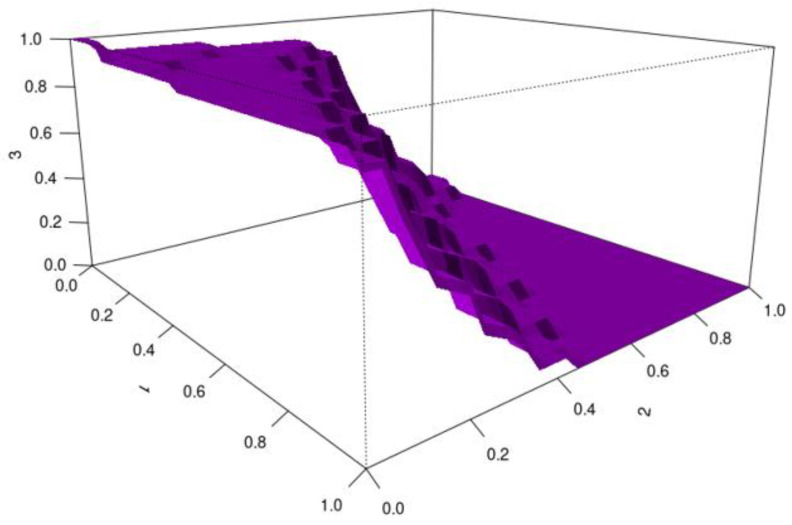
HUM image for CCL-20 (X-axis: CCL-20 quantitative values; Y-axis: HUM value).

For CCL-20, the HUM value is 0.387 and the cutoff values are 269.665–330.937. CCL-20 has been found to be an effective biomarker for distinguishing the severity of chronic hepatitis B disease. Quantitative CCL-20 values up to 269.665 indicate mild disease; values between 269.665 and 330.937 indicate moderate disease; and values above 330.937 indicate severe disease (Figure 3). 

**Figure 4:**
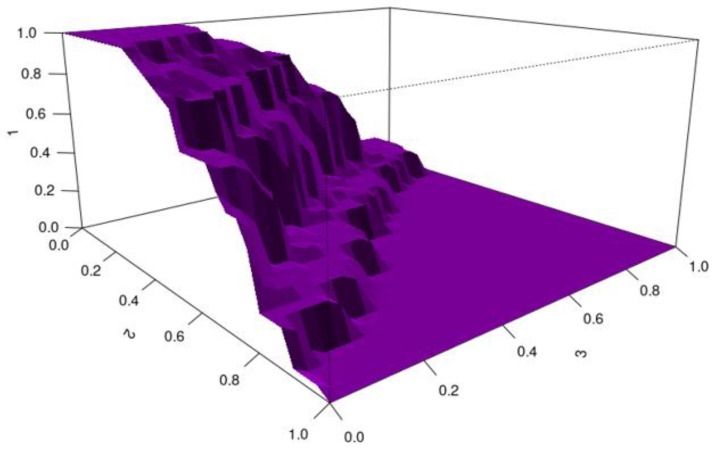
HUM image for CXCL-16 (X-axis: CXCL-16 quantitative values; Y-axis: HUM value).

For CXCL-16, the HUM value is 0.263 and the cutoff values are 2.797–9.109. CXCL-16 was found to be a relatively reliable biomarker for distinguishing the severity of chronic hepatitis B disease. Quantitative CXCL-16 values up to 2.797 indicate mild disease; values between 2.797 and 9.109 indicate moderate disease; and values of 9.109 and above indicate severe disease (Figure 4). 

**Figure 5:**
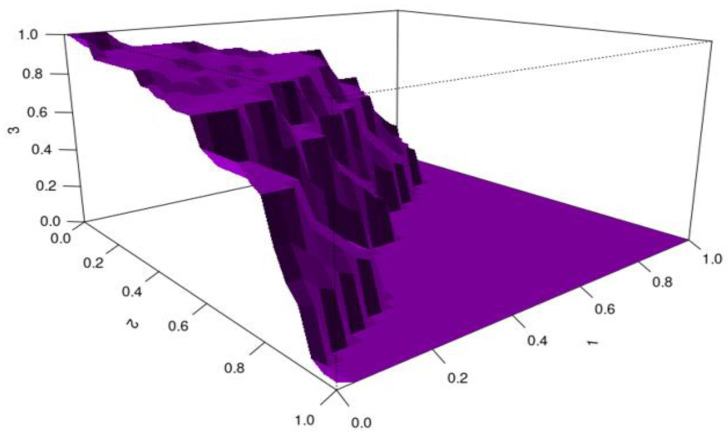
HUM image for GDF-15 (X-axis: GDF-15 quantitative values; Y-axis: HUM value).

For GDF-15, the HUM value was found to be 0.305; the cutoff values were 509.515–1051.161. GDF-15 was found to be a good biomarker for distinguishing disease severity in patients with chronic hepatitis B. Quantitative GDF-15 values up to 509.515 indicate mild disease, 509.515–1051.161 indicate moderate disease, and 1051.61 and above indicate severe disease (Figure 5). 

The AUC values of the biomarkers GDF-15 and CCL-20, which had the highest HUM values, were also found to be higher than the others. Therefore, it can be said that these two markers are more powerful when it comes to predicting chronic hepatitis B disease.

## 4. Discussion

Our study provides new evidence regarding the diagnostic and viral load-based staging capabilities of the immunological biomarkers GDF-15, CCL-20, CXCL-16, and CD8a, which could be used to manage chronic HBV infections in patients. In our study, GDF-15 was found to be “excellent” at distinguishing patients with chronic hepatitis B infection from healthy individuals, while CCL-20 was found to be “good”. Furthermore, when all the biomarkers examined in our study were evaluated using HUM analysis, a new statistical approach in this field, we found that they hold significant potential for the non-invasive staging of disease severity (defined as mild, moderate, or severe viral load). In our current study, GDF-15 was found to be a superior biomarker for diagnosing chronic hepatitis B (AUC = 0.920, with 91.7% sensitivity and 83.3% specificity), outperforming many non-invasive markers. Additionally, the strong positive correlation between GDF-15 levels and non-invasive fibrosis indices, such as viral load, APRI (AST/platelet ratio index), and GGT/platelet ratio (r = 0.31 and r = 0.23, respectively), suggests that GDF-15 may not only be a marker of inflammation but also an important indicator of ongoing liver damage and fibrosis. This confirms that GDF-15 reflects not only the presence of chronic hepatitis B disease but also its consequences, and that GDF-15 is a stress-induced cytokine [19]. Chronic hepatitis B disease involves many stress factor processes, such as the presence of viral proteins, immune attack, inflammation, and cell death [1]. In our study, we found that the GDF-15 levels increased in line with viral load, and that they correlated with markers such as AST and GGT, which indicate liver damage. This suggests that GDF-15 levels are affected by both viral load and the total pathological burden on the liver. As GDF-15 can reveal various pathological conditions, such as viral replication, inflammation, oxidative stress, hepatocyte apoptosis, and early fibrogenesis, it was found to be an excellent diagnostic biomarker for the detection of chronic hepatitis B in our study. Previous studies have also found that GDF-15 levels increase in various chronic liver diseases and are associated with the severity of fibrosis [14]. A study by Sometani et al. found that, similarly to our study, high GDF-15 levels were an independent risk factor for hepatocellular carcinoma, even in chronic hepatitis B patients receiving antiviral treatment [20].

Another study in the literature found that the AUC value for GDF-15 (AUC = 0.693) was lower than in our study for distinguishing between hepatocellular carcinoma patients and healthy individuals [21]. We believe this is related to the possibility that GDF-15 may be an immunological biomarker that enables the disease process to be detected early. In our study, statistically significantly higher CCL-20 levels were found in the patient group with a severe viral load, compared to the other groups. Together with the ability of CCL-20 to detect “good” levels of chronic hepatitis B (AUC = 0.751) and its excellent performance in staging disease severity (HUM = 0.387), this finding suggests that CCL-20 may be a dynamic biomarker of active inflammatory activity in chronic hepatitis B, with CCL-20 levels in chronic hepatitis B patients reflecting the severity of the active inflammatory response triggered by viral replication and helping to detect the necroinflammatory process of the disease. The findings of our study are consistent with the working principle of the CCL-20/CCR6 axis. CCL20 is a potent chemoattractant for all immune cells that express CCR6, including Th17 cells, which play a key role in inflammation [22]. One study of patients with alcoholic and non-alcoholic liver disease found that CCL-20 levels were significantly elevated and correlated with disease severity and fibrosis [13]. Another study by Song et al. produced similar findings to those in our study and demonstrated that CCL-20 was a highly significant immunological biomarker in the detection of HBV-induced fibrosis and chronic hepatitis B disease (AUC = 0.883) [23]. Furthermore, the negative correlation between the platelet-to-lymphocyte ratio (PLR) and CCL-20 (r = −0.27) supports the proinflammatory role of the CCL-20 biomarker; this is because a lower PLR is associated with increased inflammation. One of the most striking findings of our study is that the group with the highest levels of both CXCL-16 and CD8a was the group with a mild viral load. While these levels were significantly higher than in the control group, they did not increase further in the moderate and severe viral load groups. While these two biomarkers performed poorly overall in terms of diagnosing disease presence (AUC < 0.70), they were effective in staging disease severity. Our findings can be explained by T cell exhaustion, which is a feature of the natural history of chronic HBV infection. CXCL-16 plays a key role in recruiting CXCR6+ effector T cells (NKT and CD8+ T cells) to the liver [11]. Soluble CD8a is an important indicator of T cell activation and turnover. The high levels of both biomarkers detected in the light viral load group likely indicate an active immune response specific to the “immune clearance” phase of chronic hepatitis B disease, during which the host attempts to control the virus [24]. However, chronic hepatitis B patients experience T cell exhaustion due to continuous antigen exposure and a constantly increasing viral load [15]. Exhausted T cells lose their effector functions, proliferative capacity, and ability to produce cytokines [25]. This results in decreased overall T cell activation and turnover, which explains the higher quantitative levels of soluble CD8a and CXCL-16 detected in the group with a light viral load. A study by Wan et al. also found that CXCL-16 levels can indicate inflammation in chronic hepatitis B patients. These findings are consistent with those of our study, which also found higher levels of CXCL-16 in the chronic hepatitis B patient group than in the control group [26]. Our study also demonstrated that elevated CXCL-16 levels may be related to the viral load of the disease. The positive correlation (r = 0.25) between CXCL-16 and CD8a detected in our study strongly supports the hypothesis that quantitative levels of these two biomarkers are directly affected by a common biological process: T cell migration and activation. Our study investigated four biomarkers, providing promising results that are more comprehensive and detailed for diagnosing and staging chronic hepatitis B. Each biomarker captures a different aspect of the disease: GDF-15 (stress/injury), CCL-20 (inflammation), and CXCL-16/CD8a (T cell dynamics).

### Limitations of Our Study

While the findings of our study are promising, they have some limitations. Firstly, the study was conducted at a single center, so these findings need to be confirmed in larger, multicenter, and ethnically diverse cohorts. Furthermore, as our study only included treatment-naïve patients, the dynamics of these biomarkers during and after antiviral therapy remain unknown. One of the limitations of our study is that the BMI values of the patients were not determined. These limitations provide a clear direction for future research. Prospective, larger cohort studies with a larger patient population are necessary to confirm the value of these biomarkers in predicting disease progression, HBeAg/HBsAg seroconversion, and the development of cirrhosis or hepatocellular carcinoma. Our study will inform future research by correlating biomarker levels with liver biopsy data (necroinflammatory grades and fibrosis stages), thereby elucidating the pathophysiological roles of these molecules.

## 5. Conclusions

Our study has revealed valuable information that GDF-15, CCL-20, CXCL-16, and CD8a biomarkers, which can be measured quantitatively by a simple and cost-effective ELISA method, can be a tool that can support molecular testing (PCR) in the clinical management of chronic hepatitis B patients. GDF-15 emerged as a robust and highly accurate marker for diagnosing chronic hepatitis B, while CCL-20 was found to be a dynamic indicator of inflammation induced by the virus. Higher levels of CXCL-16 and CD8a in the low-viral-load patient group potentially provide insights into the immunological phase of the disease, distinguishing between active immune clearance and T cell exhaustion.

## Figures and Tables

**Table 1:** Study inclusion and exclusion criteria.

Inclusion Criteria	Exclusion Criteria
1. For at least six months, the diagnosis has been chronic hepatitis B with HBsAg positivity.	1. Co-infection with hepatitis C (HCV), hepatitis D (HDV), or HIV.
2. People who are already under regular medical supervision and have all their serum samples and test results on file.	2. Any history of malignancy or current malignant disease.
3. Patients who have not yet started antiviral therapy.	3. Alcohol consumption >30 g/day for men and >20 g/day for women.
4. For the control group, normal liver function and no evidence of chronic disease were observed in routine tests.	4. Other chronic liver diseases include autoimmune hepatitis, non-alcoholic fatty liver disease (NAFLD), haemochromatosis, and Wilson’s disease.
	5. Acute infection, febrile illness, or systemic inflammation within the last month.
	6. Presence of rheumatological or autoimmune diseases such as rheumatoid arthritis, systemic lupus erythematosus, inflammatory bowel diseases, etc.
	7. Type 1 or type 2 diabetes mellitus.
	8. Chronic kidney disease (glomerular filtration rate <60 mL/min/1.73 m^2^).

**Table 2:** Relationship between sociodemographic characteristics and viral load with hematological and biochemical parameters, CXCL-16, CCL-20, GDF-15, and CD8a.

Variable	Control (1) *n*= 30 ^1^	Mild Viral Load (2) *n* = 31 ^1^	Moderate Viral Load (3) *n* = 31 ^1^	Severe Viral Load (4) *n* = 34 ^1^	*p*^2^ Value
Age	46.10 ± 7.43	51.39 ± 12.85	51.23 ± 16.65	50.47 ± 18.29	0.14
Gender					0.96
Female	14 (46.67%)	15 (48.39%)	15 (48.39%)	18 (52.94%)	
Male	16 (53.33%)	16 (51.61%)	16 (51.61%)	16 (47.06%)	
Hemoglobin (g/dL)	Normal range	13.64 ± 2.41	13.26 ± 2.73	13.06 ± 3.07	0.68
Leukocyte (×10^3^/μl)	Normal range	7.37 ± 1.85	6.82 ± 1.98	8.16 ± 5.04	0.28
Platelet (×10^3^/μl)	Normal range	254.74 ± 65.87	250.58 ± 82.36	195.47 ± 86.68	*p* (2–4): 0.009 *p* (3–4): 0.017
Neutrophil (×10^3^/μl)	Normal range	4.57 ± 1.68	3.93 ± 1.56	4.40 ± 2.65	0.29
Monocyte (×10^3^/μl)	Normal range	0.65 ± 0.33	0.58 ± 0.27	0.99 ± 1.29	0.17
Lymphocyte (×10^3^/μl)	Normal range	1.97 ± 0.62	2.14 ± 0.83	2.63 ± 2.50	0.27
MPV (femtoliter;fL)	Normal range	10.38 ± 0.92	10.28 ± 0.89	10.30 ± 1.04	0.90
PDW	Normal range	12.27 ± 2.05	11.90 ± 1.70	12.15 ± 2.04	0.74
ALP (IU/L)	Normal range	89.77 ± 62.01	96.19 ± 46.92	111.18 ± 50.29	0.27
GGT (IU/L)	Normal range	20.00 (14.00–26.00)	22.00 (15.00–38.00)	44.00 (16.00–78.00)	*p* (2–4): 0.005
LDH (IU/L)	Normal range	242.48 ± 151.78	210.68 ± 97.09	247.12 ± 133.10	0.38
AST (IU/L)	Normal range	19.40 (14.00–28.10)	20.30 (15.60–26.80)	49.80 (29.20–75.00)	*p* (2–4): 0.025 *p* (3–4): 0.007
ALT (IU/L)	Normal range	19.90 (14.20–25.10)	21.50 (14.40–33.70)	65.70 (38.00–127.00)	0.235
AFP (ng/ml)	Normal range	1.82 (1.25–2.46)	2.37 (1.21–3.25)	2.14 (1.34–4.36)	0.28
CD8a (ng/mL)	14.91 (7.70–21.54)	26.58 (12.73–55.32)	21.52 (14.86–36.75)	23.05 (10.36–36.35)	*p* (1–2): 0.004
GDF-15 (pg/mL)	317.11 (236.87–378.58)	792.39 (556.81–1.496.25)	821.30 (504.99–1.407.73)	1.848.69 (1.094.06–2.721.25)	*p* (1–4): <0.001 *p* (2–4): 0.011
CXCL-16 (pg/mL)	7.31 (5.47–10.13)	12.22 (9.38–14.23)	9.17 (3.99–13.56)	8.93 (4.37–14.33)	*p* (1–2): 0.009
CCL-20 (ng/L)	75.77 (29.78–113.93)	87.46 (56.43–146.68)	195.22 (63.16–481.16)	440.00 (362.47–515.13)	*p* (1–3): <0.001 *p* (1–4): <0.001 *p* (2–3): <0.001 *p* (2–4): <0.001 *p* (3–4): 0.001
Platelet/Lymphocyte	Normal range	155.90 ± 120.34	141.44 ± 92.26	95.60 ± 58.13	*p* (2–4): 0.028
Neutrophil/Lymphocyte	Normal range	2.76 ± 1.95	2.15 ± 1.25	2.40 ± 2.74	0.34
Lymphocyte/Monocyte	Normal range	3.64 ± 1.68	3.97 ± 1.52	3.66 ± 1.83	0.67
Platelet/Neutrophil	Normal range	63.51 ± 33.80	71.67 ± 33.53	55.16 ± 29.47	0.12
MPV/Platelet	Normal range	0.04 ± 0.01	0.05 ± 0.02	0.16 ± 0.51	0.39
PDW/Platelet	Normal range	0.05 ± 0.02	0.06 ± 0.03	0.18 ± 0.61	0.44
PlateletxNeutrophil/Lymphocyte	Normal range	526.73 (418.06–772.39)	405.15 (277.50–763.51)	380.52 (177.52–546.00)	0.17
NeutrophilxMonocyte/Lymphocyte	Normal range	1.25(0.78–1.87)	0.96(0.54–1.88)	0.88(0.59–1.96)	0.12
AST/ALT	Normal range	1.19 ± 0.60	1.02 ± 0.35	0.84 ± 0.45	*p* (2–4): 0.011
Age/Platelet	Normal range	0.20 (0.15–0.27)	0.20 (0.14–0.28)	0.23 (0.12–0.41)	0.20
AST/Platelet	Normal range	0.09 (0.06–0.11)	0.09 (0.05–0.14)	0.23 (0.11–0.72)	0.13
(AST/ALT)/Platelet	Normal range	0.00 ± 0.00	0.00 ± 0.00	0.02 ± 0.07	0.40
GGT/Platelet	Normal range	0.08 (0.06–0.13)	0.10 (0.06–0.19)	0.24 (0.07–0.54)	0.10
AST/GGT	Normal range	1.02 (0.81–1.58)	0.92 (0.64–1.34)	1.37 (0.58–2.69)	0.092

^1^ Mean ± SD; n (%); median (Q1–Q3). ^2^ One-way analysis of means (not assuming equal variances); Pearson’s Chi-squared test.

**Table 3:** Sensitivity, specificity, and AUC values of CCL-20, GDF-15, CXCL-16, and CD8a for hepatitis B disease.

Biomarker	Optimum Cutting Value	AUC	Sensitivity	Specificity
GDF-15	391.908	0.920	0.917	0.833
CCL-20	183.946	0.751	0.552	0.933
CD8a	21.953	0.678	0.562	0.767
CXCL-16	11.250	0.629	0.448	0.867

**Table 4:** Correlation analysis (r values) of biomarker levels with hematological indices and non-invasive fibrosis indices.

Variable	(PlateletxNeutrophil)/Lymphocyte	Neutrophil × Monocyte/Lymphocyte	Neutrophil/Lymphocyte	AST/Platelet	GGT/Platelet
CCL-20	−0.22 *	−0.02	−0.2 *	0.24 *	0.18
CD8a	0.23 *	0.23 *	0.15	−0.12	0.06
GDF-15	0.01	0.19	0.09	0.31 **	0.23 *
CXCL-16	0.22 *	0.41 **	0.26 **	−0.01	0.08

Table Note: * *p* < 0.05, ** *p* < 0.01

## Data Availability

The datasets generated and analyzed for this study are available from the corresponding author upon request.
